# The immunostimulatory effects of retinoblastoma cell supernatant on dendritic cells

**DOI:** 10.1007/s13238-014-0029-0

**Published:** 2014-03-04

**Authors:** Juan Ma, Huamin Han, Li Ma, Changzhen Liu, Xin Xue, Pan Ma, Xiaomei Li, Hua Tao

**Affiliations:** 1 CAS Key Laboratory of Pathogenic Microbiology and Immunology, Institute of Microbiology, Chinese Academy of Sciences, Beijing, 100101 China; 2 Key Laboratory of Infection and Immunity, Institute of Biophysics, Chinese Academy of Sciences, Beijing, 100101 China; 3 Department of Obstetrics and Gynecology, China-Japan Friendship Hospital, Beijing, 100029 China; 4 Department of Immunology, Basic Medical Theory of Chinese Medicine, China Academy of Chinese Medical Sciences, Beijing, 100700 China

**Keywords:** retinoblastoma, dendritic cell, anti-tumor immunity, immunotherapy

## Abstract

Dendritic cells (DCs) are crucial for the induction and maintenance of tumor-specific immune responses. Studies have shown that tumor-associated DCs are immunosuppressed in some human tumors. However, phenotype and function of DCs in retinoblastoma (RB) remain unclear. RB cell supernatant (RBcs) was used to treat DCs *in vitro* to explore the effect of RB cells on DCs. DCs were generated from peripheral blood mononuclear cells of healthy donors. On day 5 of culture, DCs were treated with RBcs for 24 h, and then purified using magnetic beads. The maturation of DCs was induced by TNF-α or LPS. After treatment with RBcs, expression of co-stimulatory molecules CD80 and CD86 was elevated in DCs, accompanied by increased production of IL-12p70, TNF-α, IL-6, IL-1β, and IL-8 but decreased production of IL-10. RBcs neither inhibited DC maturation nor promoted DC apoptosis. Moreover, RBcs-exposed DCs stimulated allogenetic T cell proliferation and T cell-derived cytokine production. These results indicate that RBcs can improve DCs’ antigen presenting function and capability to activate T cells, suggesting that RB cells may have an immunostimulatory effect on DCs, and DC-based immunotherapy may be adopted in the treatment of RB.

## Introduction

Retinoblastoma (RB) is the most common primary intraocular malignant tumor in childhood, and the morbidity of RB is about 11 per million children below age 5 worldwide (Houston et al., 2011). The prognosis of RB patients has been dramatically improved by systematic enucleation (Khetan et al., 2013), external cryotherapy, local thermotherapy (Schueler et al., 2003), and brachytherapy (Merchant et al., 2004). Although these methods are successful at controlling the growth of the primary tumor, they cannot prevent the development of metastasis, which remains universally fatal. Moreover, there are some severe side effects related to radiotherapy or chemotherapy. Cancer immunotherapies have been generally in steady progress in this field over the past decade, particularly in the treatment of metastatic skin melanoma.

Dendritic cells (DCs) are crucial for the induction and maintenance of antitumor immune responses. Tumor-specific antigens bound to molecules of the major histocompatibility complex (MHC) on the surface of DCs are processed, then presented to and recognized by T cells. In addition, DCs provide some critical molecules, such as co-stimulatory signals and cytokines, to the T cells for their full activation. Actually, tumor-infiltrating DCs (TIDCs) are associated with prolonged survival and reduced incidence in some metastatic human tumors (Dieu-Nosjean et al., 2008; Iwamoto et al., 2003; Ladanyi et al., 2007; Nakakubo et al., 2003). However, in some other conditions, TIDCs are functionally compromised. TIDCs are phenotypically and functionally defective in colorectal cancer (Chaux et al., 1997) and melanoma (Ataera et al., 2011; Stoitzner et al., 2008), and a positive correlation of TIDCs with the poor prognosis was found in colorectal cancer (Sandel et al., 2005) and breast cancer (Treilleux et al., 2004). In hepatocellular carcinoma, circulating DCs also exhibit an immature phenotype (Beckebaum et al., 2004).

Until now, the effect of RB on human DCs has not been explored. In the present study, we used RB cell supernatant (RBcs) to mimic the tumor milieu, and performed a detailed study on the phenotype of DCs treated with RBcs. Subsequently, we investigated the effect of RBcs-exposed DCs on allogenetic T cell proliferation and cytokine production. Our study demonstrates that RBcs improves DCs’ antigen presenting function and capability to activate T cells, and DC-based immunotherapy may be adopted in the treatment of RB.

## Results

### Induction of co-stimulatory molecules CD80 and CD86 in DCs by RBcs

**Figure 1:**
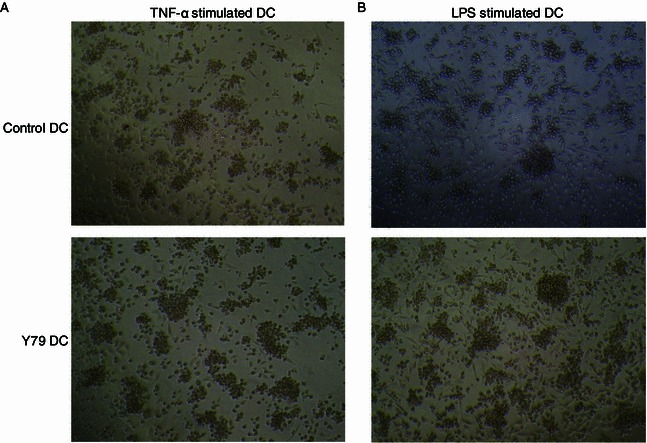
**The photomicrograph of DC cultures (200×)**. Control DCs or RBcs-exposed DCs were treated with 20 ng/mL TNF-α (A) or 1 μg/mL LPS (B) for 24 h. Y79 DC: RBcs-exposed DCs

**Figure 2:**
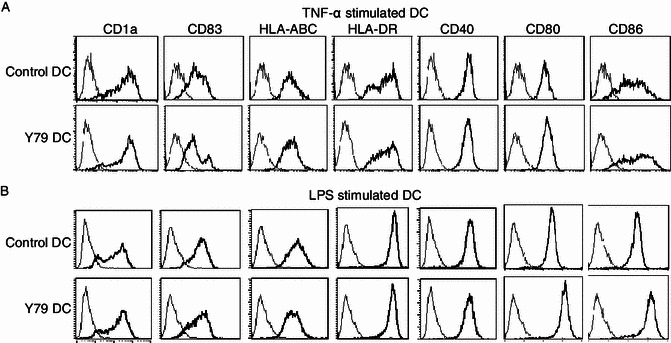
**Expression of DC markers, MHC and co-stimulatory molecules in RBcs-exposed DC**. Control DCs or RBcs-exposed DCs were treated with 20 ng/mL TNF-α (A) or 1 μg/mL LPS (B) for 24 h. The cells were then harvested for immunofluorescence staining and flow cytometry. Bold lines denote fluorescence when stained with fluorochrome-conjugated antibody to the indicated antigen, and fine lines denote fluorescence when stained with isotype control mAb. Data shown are a representative experiment of five. Y79 DC: RBcs-exposed DCs

Five-day old DCs were treated with or without RBcs for 24 h. On day 6, maturation of DCs was induced by adding 20 ng/mL TNF-α or 1 μg/mL LPS. After 24 h, all DCs appeared as big loosely adherent clumps or isolated floating cells with the typical dendritic morphology (Fig. 1). The expression of DC markers (CD1a and CD83), MHC class molecules (HLA-ABC and HLA-DR) and co-stimulatory molecules (CD40, CD80, and CD86) was determined by flow cytometry (Fig. 2). Compared with control DCs, RBcs-exposed DCs expressed higher levels of CD80 and CD86, but similar levels of CD1a, CD83, HLA-ABC, HLA-DR, and CD40. These data suggest that RB cells may enhance DCs’ capacity in priming T cell responses, whereas have no effect on the maturation of DCs.

### Induction of IL-12p70, TNF-α, IL-6, IL-1β, IL-8 and inhibition of IL-10 in DCs by RBcs

**Figure 3:**
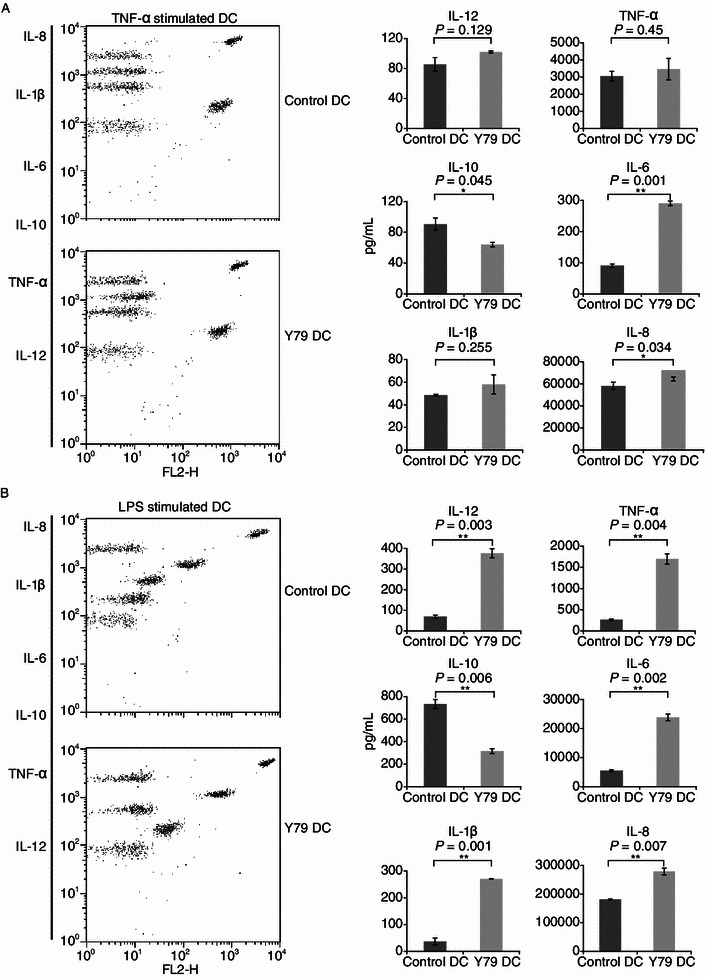
**Production of cytokines IL-12, IL-10, TNF-α, IL-1β, IL-6, and IL-8 by RBcs-exposed DCs**. Control DCs or RBcs-exposed DCs (4 × 10^4^ cells/well) were treated with 20 ng/mL TNF-α (A) or 1 μg/mL LPS (B) for 24 h. Concentrations of cytokines in cell-free supernatants were analyzed by CBA Human Inflammation Kit that could identify all six kinds of cytokines in a single sample. Quantitative production of a particular kind of cytokine is indicated by PE-labeled-specific Ab staining. The data are mean values ± SD of triplicate determinations. Data shown are a representative experiment of three. *P < 0.05, vs. control DCs. **P < 0.01, vs. control DCs. Y79 DC: RBcs-exposed DCs

In addition to co-stimulatory molecules, DC-derived cytokines also play an important role in priming T cell response. Cytokine production in DCs was assayed using CBA Human Inflammation Kit. Compared with control DCs, RBcs-exposed DCs secreted more IL-12p70, TNF-α, IL-6, IL-1β, and IL-8, but less IL-10, a potent immunosuppressive cytokine (Fig. 3). Moreover, alterations in cytokine production induced by RBcs in LPS-matured DCs were more significant than those in TNF-α-matured DCs (Fig. 3B). These results further indicate that RB cells can increase DCs’ capacity to activate T cells.

### No effect of RBcs on DC apoptosis

**Figure 4:**
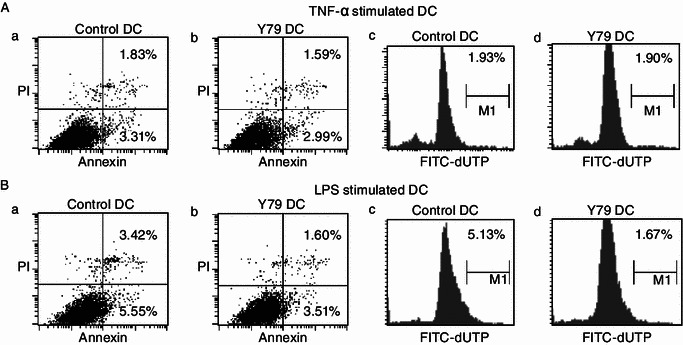
**Apoptosis of RBcs-exposed DCs**. Control DCs or RBcs-exposed DCs were treated with 20 ng/mL TNF-α (A) or 1 μg/mL LPS (B) for 24 h. DCs were stained with FITC-Annexin-V and propidium iodide (PI), and the proportion of apoptotic cells (Annexin^+^/PI^-^) was determined by flow cytometry (a and b). Apoptosis was also assessed by a FACS-based TUNEL assay utilizing FITC-dUTP. The proportion of apoptotic cells (dUTP^+^) was determined by flow cytometry (c and d). Data shown are one representative experiment of three. Y79 DC: RBcs-exposed DCs

As tumor cells can escape immune destruction by inducing DC apoptosis (Kanto et al., 2001; Kiertscher et al., 2000; Ma et al., 2010), we evaluated whether RB cells could affect DC apoptosis (Fig. 4). FITC-Annexin-V and propidium iodide (PI) were used to stain DCs, and the proportion of apoptotic cells (Annexin^+^/PI^-^) was determined by flow cytometry. No significant difference in apoptotic proportion was observed between control DCs and RBcs-exposed DCs. DC apoptosis was further assessed by a FACS-based TUNEL assay utilizing FITC-dUTP. It was found that the apoptotic rate of RBcs-exposed DCs was similar to that of control DCs. These data indicate that RB cells have no effect on DC apoptosis.

### Stimulation of T cell proliferation by RBcs-exposed DCs

**Figure 5:**
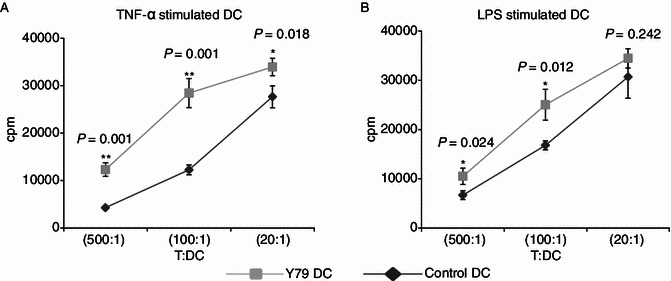
**Enhanced allogenetic T cell proliferation stimulated by RBcs-exposed DCs**. After treatment with TNF-α (A) or LPS (B) for 24 h, varying numbers (400, 2000, or 10000) of irradiated (30 Gy) DCs were added to triplicate wells containing 2 × 10^5^ purified allogeneic T lymphocytes and incubated for 4 days. Cultures were pulsed during the final 8 h of incubation, and incorporation of [^3^H]-thymidine was measured. The data are mean values ± SD of triplicate determinations. Data shown are a representative experiment of three. *P < 0.05, vs control DCs. **P < 0.01, vs control DCs. Y79 DC: RBcs-exposed DCs

To verify the immunostimulatory effect of RBcs-exposed DCs, DCs treated with or without RBcs were irradiated and then used to stimulate purified allogeneic T cells (2 × 10^5^ per well). Proliferation of T cells was analyzed by measuring the incorporation of [^3^H]-thymidine. As shown in Fig. 5, a significant increase in T cell proliferation was observed when they were co-cultured with RBcs-exposed DCs at T/DC ratio of 500:1 and 100:1, indicating that DCs’ capacity to activate T cells was enhanced after treatment with RBcs.

### Induction of T cell-derived cytokines by RBcs-exposed DCs

**Figure 6:**
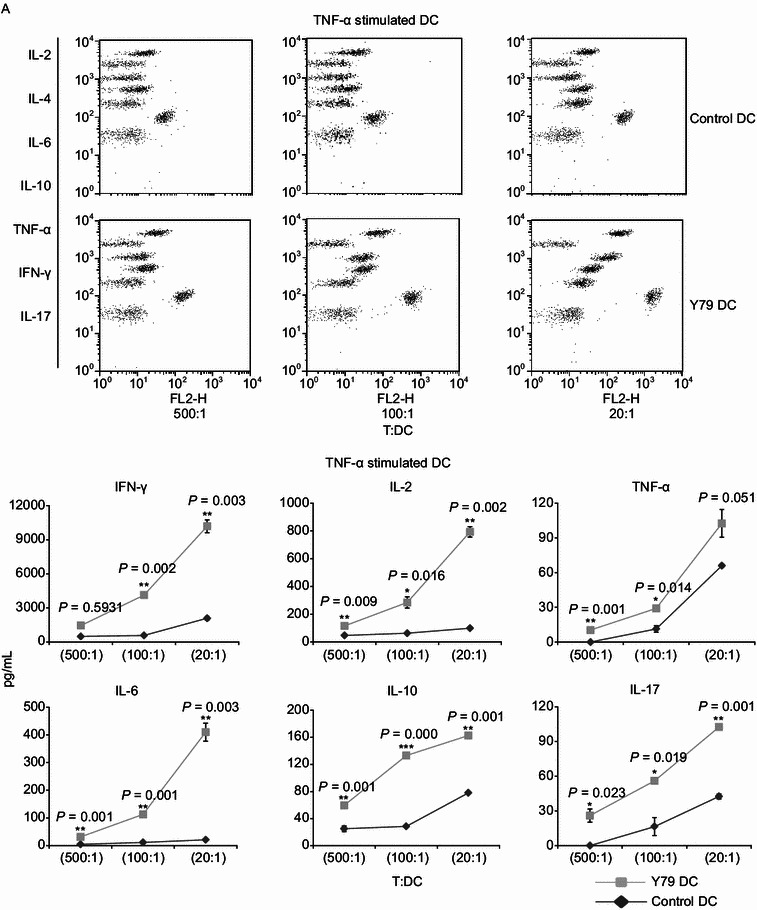

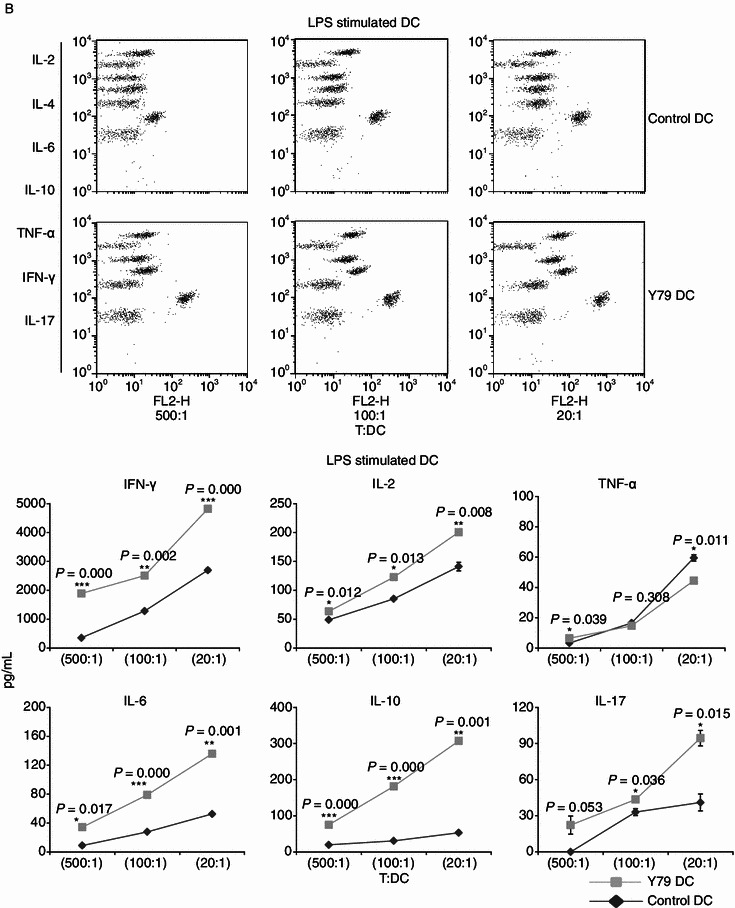
**Increased cytokine production of by allogenetic T cell stimulated with RBcs-exposed DCs**. After treatment with TNF-α (A) or LPS (B) for 24 h, varying numbers (400, 2000, or 10000) of irradiated (30 Gy) DCs were added to triplicate wells containing 2 × 10^5^ purified allogeneic T lymphocytes and incubated for 3 days. The concentrations of cytokines producted in cell-free supernatants were analyzed by CBA Human Th1/Th2/Th17 Cytokine Kit that could identify all seven kinds of cytokines in a single sample. Quantitative production of a particular kind of cytokine is indicated by PE-labeled-specific Ab staining. The data are mean values ± SD of triplicate determinations. Data shown are a representative experiment of three. *P < 0.05, vs. control DCs. **P < 0.01, vs. control DCs. ***P < 0.001, vs. control DCs. Y79 DC: RBcs-exposed DCs

To further confirm the immunostimulatory effect of RBcs-exposed DCs, levels of T cell-derived cytokines were measured in above T-DC co-culture system using the CBA human Th1/Th2/Th17 cytokine kit. It was observed that RBcs-exposed DCs stimulated T cells to secrete much more cytokines than control DCs at different T/DC ratio, including IFN-γ, IL-2, TNF-α, IL-6, IL-10, and IL-17 (Fig. 6). On the contrary, no IL-4 production was found in T cells treated with either control DCs or RBcs-exposed DCs. These data indicate that RBcs-exposed DCs induce T cells to produce Th1 and Th17 cytokines predominantly.

## Discussion

DCs play a critical role in tumor immune surveillance by initiating tumor-specific immune responses. Clinically, infiltration of DCs is correlated with a better prognosis in various human tumors (Dieu-Nosjean et al., 2008; Iwamoto et al., 2003; Ladanyi et al., 2007; Nakakubo et al., 2003), which is attributed to inhibition of tumor growth and metastasis by DCs (Lim et al., 2007; Movassagh et al., 2004; Preynat-Seauve et al., 2007). However, the immune function of DCs is sometimes suppressed due to some factors produced by tumors, such as IL-10, transforming growth factor-β (TGF-β), vascular endothelial growth factor (VEGF), IL-6, macrophage colony-stimulating factor (M-CSF), and prostaglandin E_2_ (PGE_2_) (Preynat-Seauve et al., 2006; Zou, 2005). The molecular mechanisms of DC dysfunction may be related to activation of the signal transducers and activators of transcription 3 (STAT3) and inhibition of nuclear factor kappa-B (NF-κB) (Gottfried et al., 2008; Wang et al., 2004).

Until now, the role of DCs in RB has not been explored, and the effect of RB on DCs remains unknown. In the present study, we first identified the alterations in the phenotype of DCs after treatment with RBcs. It was found that RBcs did not inhibit the maturation of DCs, based on the finding that RBcs had no effect on the expression of DC markers CD1a and CD83. Our observation is inconsistent with some other studies in which maturation of DCs is suppressed by tumor cells or tumor condition media (Bharadwaj et al., 2007; Gabrilovich et al., 1996; Ma et al., 2010; Michielsen et al., 2011; Sombroek et al., 2002), suggesting that inhibition of DCs’ maturation may be tumor-specific. Because some tumor cells can induce DC apoptosis to escape immune surveillance (Kanto et al., 2001; Kiertscher et al., 2000; Ma et al., 2010), we evaluated the effect of RBcs on DC apoptosis. As shown in the present study, RBcs failed to induce DC apoptosis. There is one report showing that mature DCs can upregulate Bcl-X to resist Fas/CD95-mediated DC apoptosis (Lundqvist et al., 2002).

Our study revealed that RBcs upregulated co-stimulatory molecules CD80 and CD86 in DCs. It has been well established that these molecules are required for optimal T cell activation via binding their receptors CD28 and CD152 (CTLA4) on T cells (Carreno and Collins, 2002; Collins et al., 2005). Therefore, upregulation of CD80 and CD86 by RBcs suggests that RB cells may enhance T cell immune response through DCs. Consistent with this finding, an increase in T cell proliferation was observed when T cells were co-cultured with RBcs-exposed DCs in the present study. In addition, these T cells were found to produce more cytokines than control RBcs-untreated DCs. These results indicate that RB cells improve DCs’ capacity to activate T cell, even though phenotype and function of DCs in RB tumor microenvironment still need to be further identified.

In addition to co-stimulatory molecules CD80 and CD86, more cytokines were produced by RBcs-exposed DCs, such as IL-12, an essential cytokine stimulating Th1 responses. Consistent with IL-12 overproduction, RBcs-exposed DCs induced T cells to secrete more IFN-γ. Interestingly, it was found that CD80/86 was synergistic with IL-12 for inducing T cell proliferation and IFN-γ production (Kubin et al., 1994). Some cancer models have demonstrated the anti-tumor activities of IL-12 (Colombo and Trinchieri, 2002). Endogenous IL-12 is required for resistance to transplantable tumors or chemically induced tumors, and recombinant IL-12 treatment inhibits tumor establishment or induces tumor regression (Colombo and Trinchieri, 2002). The anti-tumor mechanisms of IL-12 may also be related to other cytotoxic lymphocytes, such as NK cells, whose maturation and activation are also dependent on IL-12 (Loza and Perussia, 2001).

Contrary to upregulation of IL-12, IL-10 was downregulated in RBcs-exposed DCs. As an immunosuppressor, IL-10 inhibits the expression of MHC class molecules and co-stimulatory molecules, and prevents the production of Th1 cytokines IL-2 and IFN-γ by antigen-presenting cells (APCs). Therefore, IL-10 favors tumor evasion from immune surveillance via suppressing antigen presentation and T cell activation (Lippitz, 2013). IL-10 is overexpressed in a series of human cancers, which is associated with advanced stage and bad prognosis (Lippitz, 2013). Our study shows that RB cells may trigger immune responses by reducing IL-10 production, in addition to increasing IL-12 secretion by DCs.

In summary, we for the first time reveal that RBcs can enhance DCs’ capacity to activate T cells based on the following findings: First, RBcs upregulated the expression of co-stimulatory molecules CD80 and CD86 in DCs. Second, RBcs induced the production of IL-12p70, TNF-α, IL-6, IL-1β, and IL-8, whereas reduced the secretion of IL-10 by DCs. Third, RBcs-exposed DCs stimulated T cell proliferation. Finally, RBcs-exposed DCs induced T cells to release Th1/Th17 cytokines predominantly. These results suggest that RB cells may have an immunostimulatory effect on DCs, and immunotherapy aimed at DCs may be a potential way to treat RB.

## Materials and methods

### RB cell line and DCs

RB cell line Y79 was obtained from ATCC (HTB-18; Rockville, MD), and maintained in RPMI-1640 medium supplemented with 10 mmol/L HEPES, 2 mmol/L L-glutamine, 100 U/mL penicillin, 100 μg/mL streptomycin, and 10% heat-inactivated fetal calf serum (FCS, Sigma Chemical Co., St. Louis, MO, USA). Peripheral blood mononuclear cells (PBMCs) were isolated by Ficoll-Hypaque density gradient centrifugation from healthy individuals. To generate DCs, the mononuclear cell fraction was washed twice with RPMI-1640, suspended in RPMI-1640 at 2.5 × 10^6^ cells/mL, and seeded in a 6-well plate (Becton) at 2 mL per well. The plate was incubated at 37°C for 2 h, and the non-adherent cells were discarded. The adherent cells were cultured for 6 days in 2.5 mL RPMI supplemented with 10 mmol/L HEPES, 2 mmol/L L-glutamine, 100 U/mL penicillin, 100 μg/mL streptomycin, 10% heat-inactivated FCS, 50 ng/mL rhGM-CSF, and 20 ng/mL rhIL-4 (R&D System, Minneapolis, MN). Half-volume medium exchange was performed every 3 days with medium containing fresh cytokines.

### Treatment of DCs with RBcs

RBcs was prepared by seeding 10-cm dish (Falcon; BD Bioscience, Franklin Lakes, NJ, USA) with 1 × 10^7^ RB cell in 10 mL of completed medium for 24 h and centrifuged to remove cell debris. On day 5 of DC culture, RBcs was added to test DCs, while the same volume of culture medium was added to control DCs. On day 6, maturation of DCs was induced by adding 20 ng/mL of TNF-α (R&D System) or 1 μg/mL of LPS (Sigma), and the phenotype of DCs was determined by flow cytometry after incubating with TNF-α or LPS for 24 h. In some experiments, before adding of TNF-α or LPS, DCs were purified on day 6. Briefly, RBcs-treated DCs or control DCs were washed extensively and then purified with microbeads on auto-MACS columns using a Blood Dendritic Cell Isolation kit (Miltenyi Biotech, BergischGladbach, Germany) according to the manufacturer’s instructions. In short, isolation of DCs was performed in a two-step procedure. First, cells labeled with the Non-DC Depletion Cocktail comprising with CD14 and CD19 magnetic beads were depleted. Then DCs were positively selected by labeled with DC Enrichment Cocktail comprising with CD1c (BDCA-1), CD304 (BDCA-4/Neuropilin-1), and CD141 (BDCA-3) magnetic beads. Purified DCs samples contained >95% CD1c^+^ DCs as evaluated by the Blood Dendritic Cell Enumeration Kit (Miltenyi Biotech).

### Flow cytometry and Reagents

The following monoclonal antibodies (mAb) conjugated with either fluorescein isothiocyanate (FITC) or phycoerythrin (PE) were used: CD1a, CD83, CD40, CD80, CD86, HLA-ABC, and HLA-DR (BD Pharmingen, San Diego, CA). Mouse IgG isotype control mAbs were purchased from eBioscience (San Diego, CA). To examine apoptosis, DCs were stained by FITC-Annexin-V and propidium iodide (PI) or assessed by a FACS-based TUNEL assay (APO-DIRECTTM Kit; BD Pharmingen). Samples were analyzed using a flow cytometer (FACSCalibur; BD Bioscience) and data were processed using the accompanying software (CellQuest; BD Bioscience).

### DC-derived cytokine assays

Purified DCs were cultured in a flat-bottom 96-well micro-culture plate at a density of 4 × 10^4^ cells/well in 0.2 mL of culture medium, in the presence of the TNF-α or LPS. Cell free supernatant was collected 24 h later and levels of IL-12p70, TNF-α, IL-10, IL-6, IL-1β, and IL-8 were measured by Cytometric Bead Array (CBA) assay with the Human Inflammation Kit (BD Bioscience) according to the protocols recommended by the manufacturer.

### T cell proliferation assay

Heparinized blood samples were obtained from healthy individuals and PBMCs were isolated by Ficoll-Hypaque centrifugation. CD3^+^ T cells were purified with microbeads on auto-MACS columns using a pan T cell Isolation kit (Miltenyi Biotech) according to the manufacturer’s instructions. Purified CD3^+^ T cell samples contained >98% CD3^+^ T cells as determined by flow cytometry. DCs were irradiated with an X-irradiator (Gammacell 40 Exactor; MDS Nordion International, Inc., Ottawa, Ontario, CA) at 30G. Purified allogeneic CD3^+^ T cell seeded into a round-bottom 96-well micro-culture plate at 2.0 × 10^5^ per well were co-cultured with purified, stimulated, irradiated DCs at T:DC ratio of 20:1, 100:1, or 500:1. All experiments were done in triplicate, and T cells alone were used as the background control. The cultures were incubated for 4 days at 37°C in 5% CO_2_ in air, pulsed with [^3^H]-thymidine (1.0 μCi/10 μL/well) during the last 8 h of incubation, and then harvested onto glass filters with an automated cell harvester. Radioactivity was assessed by liquid scintillation spectrometry (Tomtec, Orange, CT), and expressed as counts per minute.

### T cell-derived cytokine assays

Cell free supernatant was collected from T cell-DC co-culture system 72 h later and levels of IFN-γ, TNF-α, IL-10, IL-6, IL-4, IL-2, and IL-17 were measured by CBA assay with the Human Th1/Th2/Th17 Kit (BD Bioscience) according to the protocols recommended by the manufacturer.

### Statistical analyses and reproducibility

Experiments were repeated at least twice and usually three times. Results were expressed as mean ± SD. Statistical analyses were performed by independent *t*-test using the computer software PEMS 3.1 for Windows software (Package for Encyclopaedia of Medical Statistics, Chengdu, China) or PASW Statistics 18. *P* < 0.05 was considered significant. Significance was denoted by an asterisk in the figures.

## Abbreviations

APCs: antigen-presenting cells
DCs: Dendritic cells
mAb: monoclonal antibodies
M-CSF: macrophage colony-stimulating factor
MHC: major histocompatibility complex
NF-κB: nuclear factor kappa-B
PBMCs: peripheral blood mononuclear cells
RB: retinoblastoma
RBcs: RB cell supernatant
STAT3: signal transducers and activators of transcription 3
TGF-β: transforming growth factor-β
VEGF: vascular endothelial growth factor
